# Paradoxical Psychology

**Published:** 1860-01-01

**Authors:** 


### THE JOURNAL

OF

### PSYCHOLOGICAL MEDICINE

AND

### MENTAL PATHOLOGY.

JANUARY 1, 1860.

### Art. I.—PARADOXICAL PSYCHOLOGY.

It is an old story, and one that has often been dwelt upon, that genius and insanity are near akin. Both philosophers and poets have told the story, the former having ballasted it with their authority, and the latter bedecked it with their fancy. The multitude have also their version, and the belief which underlies it, crops out in the proverbs, or is implied in the superstitions of almost every race under the sun.

The vernacular hath it that *Fools and philosophers were made out of the same metal;* the term *fool* being applicable either in its primitive and technical, or in its ordinary and conventional sense. A living writer* expresses the sentiment of the proverb by the wicked remark that "philosophers are often but ingenious lunatics." The popular belief regarding the consanguinity of insanity and poetic genius, has been very happily represented by our greatest dramatist. He tells us that—

> "The lunatic, the lover, and the *poet*,
> Are of imagination all compact!
> One has more devils than vast hell can hold;
> That is the madman: the lover all as frantic,
> Sees Helen's beauty in a brow of Egypt:
> The poet's eye, in a fine frenzy rolling,
> Doth glance from heaven to earth, from earth to heaven;
> And as imagination bodies forth
> The form of things unknown, the poet's pen
> Turns them to shapes, and gives to airy nothings
> A local habitation and a name."†

* Bulwer,—*My Novel.*

† *Midsummer Night's Dream,* act v. sc. 1.

He of whom Shakespeare himself has written—

——"whose deep conceit is such,
As passing all conceit, needs no defence"—

Spenser, personified imagination as one who "could things to come foresee;" and he describes this personification in terms perfectly consistent with the notions which the great dramatist assigns to fancy, in the genesis of lunacy, poesy, and love. We read in the *Faerie Queene* how Sir Guyon, for his instruction, was shown Imagination in the House of Temperance. The Elfin Knight beheld the embodied power inhabiting a chamber within which were depicted in sundry colours the many exaggerated feats of an erratic fancy—

"Infernall hags, centaurs, fiendes, hippodames,
Apes, lyons, eagles, owls, fooles, lovers, children, dames.

And all the chamber filled was with flyes,
Which buzzed all about, and made such sound,
That they encombred all men's eares and eyes,
Like many swarmes of bees assembled round,
After their hives with honey do abound.
All those were idle thoughtes and fantasies,
Devices, dreams, opinions unsound,
Themes, visions, sooth-sayes, and prophesies,
And all that fained is—as leasings, tales and lies.

Emongst them all sate which wonned there,
That hight Phantastes by his nature true;
A man of yeares, yet fresh, as mote appeare,
Of swarth complexion, and of crabbed hew,
That him full of meláncholy did shew;
Bent, hollow, beetle brows, sharpe staring eyes,
That mad or foolish seemed; one by his vise
Mote deeme him borne with ill-disposed skyes,
When oblique Saturne sat in the house of agonyes."*

Ebenezer Sibley hath written in his ponderous quarto† on the *Occult Sciences*, that "All melancholy and nervous affections, quartan agues, falling sickness, black-jaundice, tooth-ache," &c., are under the government of Saturn.

The introduction by Shakespeare of the lover into the category of kinship between genius and insanity is true to the spirit of the idea, and has its warranty even in the letter. Thus we learn from Chaucer's exquisite description of the love-stricken Arcite that—

——"In his gaze, for all the world he ferd
Nought only like the lovers maladie
Of Ereos, but rather ylike manie,‡
Engendred of humourous melancolike,

* Bk. ii. canto ix. st. l., li., lii. † 1790, p. 108. ‡ Madness.

Beforne his hed in his calle fantastike.
And shortly turned was all up so down,
Both habit and eke dispositiones
Of him, this woful lover, Dan Arcite."*

Plato speaks of love as a divine madness, and he represents Socrates as combating, in the recantation of that philosopher concerning love,† the falsity of the assertion, "which declares that when a lover is present, favour ought to be shown to one who is not in love, because the one is mad, and the other in his sober senses."

The supernatural character which was originally believed to appertain to genius is retained in the name, and the heaven-born gift, whether proceeding from the genius of prophecy, of poetry, of science, or of love—whether from Apollo, or the Muses, or Cupid—was thought to be inextricably linked with madness. Hence from the notion that genius was the manifestation of a divine afflatus came first the startling paradox that madness was not an evil, but a blessing.

Plato in the recantation just spoken of instructs us thus:—

"If it were universally true that madness is evil, the assertion [that we should neglect the lover because he is mad] would be correct. But now the greatest blessings we have spring from madness, when granted by divine bounty. For the prophetess at Delphi, and the priestesses at Dodona, have, when mad, done many and noble services for Greece, both privately and publicly, but in their sober senses little or nothing. And if we were to speak of the sybil and others, who, employing prophetic inspiration, have correctly predicted many things to many persons respecting the future, we should be too prolix, in relating what is known to every one. This, however, deserves to be adduced by way of testimony, that such of the ancients as gave names to things, did not consider madness as disgraceful or a cause of reproach; for they would not have attached this very name to that most noble art by which the future is discerned, and have called it a mad art, but considering it noble when it happens by the divine decree, they gave it this name; but the men of the present day, by ignorantly inserting the letter τ, have called it the prophetic art. [μανια is *madness*, μανική, *the mad art*, μαντική, *the prophetic art.*] Since also with respect to the investigation of the future by people in their senses, which is made by means of birds and other signs, inasmuch as men, by means of reflection, furnished themselves by human thought with intelligence and information, they gave it the name of prognostication, which the moderns, by using the emphatic long ω, now called augury. [οἰονιστική, *prognostication*, οἰωνιστική, *augury*]. But how much more perfect and valuable then prophecy is than augury, one name than the other, and one effect than the other, by so much did the ancients testify that madness is more noble than sound sense, that which comes from God than that

* *Knightes Tale.* † *Phædrus.*

which proceeds from man. Moreover, for those dire diseases and afflictions which continued in some families in consequence of ancient crimes committed by some or other of them, madness springing up and prophesying to those to whom it was proper, discovered a remedy, fleeing for refuge to prayers and services of the gods, whence obtaining purifications and atoning rites, it made him who possessed it sound, both for the present and the future, by discovering to him who was rightly mad and possessed, a release from present evils. There is a third possession and madness proceeding from the Muses, which seizing upon a tender and chaste soul, and rousing and inspiring it to the composition of odes and other species of poetry, by adorning the countless deeds of antiquity, instructs posterity. But he who without the madness of the Muses approaches the gates of poesy under the persuasion that by means of art he can become an efficient poet, both himself fails in his purpose, and his poetry, being that of a sane man, is thrown into the shade by the poetry of such as are mad."*

It is curious and most instructive to observe how the double paradox, contained in the foregoing paragraph from the *Phædrus*—firstly, that madness is more noble than sound sense; secondly, that so far from being an evil it was in some instances the means of release from evil—has been preserved in its essential character from the time of Plato even to the present time.

For our purpose it is not necessary that we should trace the history of the paradox during the period named, with any degree of minuteness. It will be sufficient for us to show its existence at an intermediate date, and now.

Not every form of insanity was deemed a blessing by Plato, but only such forms as were supposed to be occasioned by the influence of beneficent deities. Hence the philosopher's notions of the evil or good of madness were entirely governed by the mythological ideas of the period in which he lived. Change the form of belief, and we find precisely the same conceptions respecting the nature of madness to have existed among the Christian communities of the middle ages. When the delusions of the insane or the fever-stricken, or the dreams of the ascetic, took a form consistent with the dogmas of the Church, they were hailed as the sure tokens of divine inspiration; when the reverse, as the promptings of the devil. Church history abounds with illustrations of the truthfulness of this opinion.

The Venerable Bede tells us of the holy man Fursey, who "fell into some infirmity of body, and was thought worthy to see a vision from God." This holy man, who lived about A.D. 653, was favoured with certain apocalyptic dreams, and the historian further informs us in regard to him, that "An ancient brother of our monastery is still living, who is wont to declare that a very

* *Phædrus.* Cary's Trans.

sincere and religious man told him that he had seen Fursey himself in the province of the East Angles, and heard those visions from his mouth; adding, that though it was a most sharp winter weather, and a hard frost, and the man was sitting in a thin garment when he related it, yet he sweated as if it had been in the greatest heat of summer, *either through excessive fear, or spiritual consolation*."*

Bede also recounts, among other examples of prophetic power, two instances which occurred, one in a child, the other in a nun, at the point of death.

In the monastery of Barking (A.D. 676) there was a little boy named Esica, who was about three years of age. This child was seized with pestilence, and when dying he called thrice upon one of the consecrated virgins in the monastery, "directing his words to her by her own name, as if she had been present, Eadgith! Eadgith! Eadgith! and thus ending his temporal life, entered into that which was eternal. The virgin whom he called, was immediately seized, where she was, with the same distemper, and departing this life the same day on which she had been called, followed him that called her into the heavenly country."†

One of the nuns in the same monastery, being also seized with pestilence, and reduced to extremity, suddenly began about midnight to cry out to those who attended her, requesting them to extinguish the candle that was lighted there; but no one heeded her. Whereupon she said, "'I know you think I speak this in a raving fit, but let me inform you it is not so; for I tell you, that I see this house filled with so much light, that your candle seems to me to be dark.' And when still no one regarded what she said, or ventured any answer, she added, 'Let that candle burn as long as you will; but take notice, that it is not my light, for my light will come to me at the dawn of day.' Then she began to tell, that a certain man of God, who had died that same year, had appeared to her, telling her that at the break of day she should depart to the heavenly light. The truth of which vision was made out by the virgin dying as soon as the day appeared."‡

Still more to our purpose is the account which the venerable historian gives of the development of poetic genius in the Anglo-Saxon poet Cædmon, a brother of the monastery of Streaneshalch (Whitby—A.D. 680). "He was wont," writes Bede, "to make pious and religious verses, so that whatever was interpreted to him out of Scripture, he soon after put the same into poetical expressions of much sweetness and humility, in English,

* *Ecclesiastical History of England*, bk. iii. ch. 19. Dr. Giles's Ed.
† Bede, *Eccles. Hist.* bk. iv. ch. 8. ‡ *Ib.*

which was his native language. By his verses the minds of many were often excited to despise the world, and to aspire to heaven. Others after him attempted, in the English nation, to compose religious poems; but none could ever compare with him, for he did not learn the art of poetry from man, but from God."

This last sentence is a Christianized form of one of Plato's remarks already quoted from the *Phœdrus*, to the effect that he who assays the poetic art without being possessed of the divine madness of the Muses, will fail in his efforts, and his poetry, being that of a sane man, will be greatly inferior to that of one who is mad.

Cædmon, it would appear, on account of the source of his gift, was never able to compose "any trivial or vain poem." Sacred themes alone "suited his religious tongue." He had lived in a secular habit until he was far advanced in life, and occasionally was present at entertainments where it was customary, in order to promote mirth, for each guest to sing in succession. But Cædmon, having never learnt anything of versifying, used when the instrument with which the songs were accompanied approached him, to rise up from the table and return home.

"Having done so at a certain time, and gone out of the house where the entertainment was, to the stable, where he had to take care of the horses that night, he there composed himself to rest at the proper time; a person appeared to him in his sleep, and saluting him by his name, said, 'Cædmon, sing some song to me.' He answered, 'I cannot sing; for that was the reason why I left the entertainment, and retired to this place, because I could not sing.' The other who talked to him, replied, 'However, you shall sing.' 'What shall I sing?' rejoined he. 'Sing the beginning of created beings,' said the other. Hereupon he presently began to sing verses to the praise of God, which he had never heard. . . . . Awaking from his sleep, he remembered all that he had sung in his dream, and soon added much more to the same effect in verse worthy of the Deity.

"In the morning he came to the steward, his superior, and having acquainted him with the gift he had received, was conducted to the abbess, by whom he was ordered, in the presence of many learned men, to tell his dream, and repeat the verses, that they might all give their judgment what it was, and whence his verse proceeded. They all concluded that heavenly grace had been conferred on him by our Lord. They expounded to him a passage in Holy Writ, either historical or doctrinal, ordering him, if he could, to put the same into verse. Having undertaken it, he went away, and returning the next morning, gave it to them composed in most excellent verse; whereupon the abbess, embracing the grace of God in the man, instructed him to quit the secular habit, and take upon him the monastic life; which being accordingly done, she associated him to the rest of the brethren in her monastery, and ordered that he should be taught the whole series of sacred history."*

In the fourteenth century, among many mystical enthusiasts Suso is particularly noteworthy. He, it is recorded, was called to a spiritual life by the *Eternal Wisdom* manifesting itself to him in the form of a maiden bright as the sun. In order to attain the highest sanctity, he submitted himself to most severe torture, and he was rewarded by the *Holy Child* appearing to him, and putting to his lips a vessel of spring water. At another time the *Blessed Virgin* gave him a draught from her own heart. Encouraged by these manifestations of divine favour, he persisted in a life of self-torture. At one time he wore constantly, night and day, a close-fitting shirt, in which had been fixed one hundred and fifty nails, the points turned inwards towards the flesh; and lest at any time he should be tempted to relieve himself, he clad his hands with gloves which were covered with sharp blades. At another time he carried between his shoulders a wooden cross perforated by thirty nails, the points of which rested against the skin. He pursued this system of mortification from his eighteenth to his fortieth year, and its gratefulness to the Divine Power was manifested by numerous heavenly visions and other instances of divine favour. He was permitted to hear the angelic host hymn the praises of the Highest, and often he has been comforted by angels, and been led by them in the spirit to join the celestial dance. "One day, when thus surrounded in a vision, he asked a shining prince of heaven to show him the mode in which God had his secret dwelling in his soul. Then answered the angel—'Take a gladsome look into thine inmost, and see how God in thy living soul playeth his play of love.' Straightway I looked, and behold the body about my heart was as clear as crystal, and I saw the Eternal Wisdom calmly sitting in my heart in lovely wise,† and close by that form of beauty, my soul, leaning on God, embraced by him, and pressed to his heart, full of heavenly longing, transported, intoxicated with love."‡

Suso declares that he wrote his *Horologe of Wisdom, or Book of the Eternal Wisdom,* which he finished in 1340, from inspiration; he himself being "ignorant and passive, but under the immediate impulse and illumination of the Divine Wisdom."

At a period still less remote from us we find, among a host of canonized individuals, St. Catherine of Siena, whose holy life

* *Eccles. Hist.* bk. iv. ch. 24.

† "It seemed to me that my body melted away, and became transparent. I saw very clearly within my breast the hachisch that I had eaten, under the form of an emerald, which emitted millions of little sparks."—*Moreau* (*de Tours*) *du Hachisch,* p. 21.

‡ *Hours with the Mystics.* By R. A. Vaughan, B.A., vol. i. p. 290.

commenced with visions when she was but six years of age, and who was solemnly betrothed to our Lord not long after. "She is said to have shown a purity and inspiration in her poems which might have ranked her with Dante and Petrarch. Here is divine inspiration—holy and miraculous power !"*

St. Hildegarde may be cited as another example. She stands conspicuous among the canonized from the numerous visions with which she was favoured. As in the case of St. Catherine of Siena, the visions of Hildegarde commenced in childhood. "In the third year of my life," she tells us, in a letter to the monk Wibertus, "I beheld such a light that my soul trembled; but, on account of my youth, I was unable to describe it. In my eighth year I was admitted to spiritual communion with God; and, till I was fifteen, I beheld many visions, which I related in my simplicity, and those who heard me were astonished, wondering from whence they could come. At that time I also felt surprised that while I saw internally with my soul, I also saw outwardly with my eyes; and as I never heard of a similar thing in others, I endeavoured to conceal my visions as much as possible. Many things of the world remained unknown to me on account of my continual ill-health, which, dating from my birth, weakened my body and destroyed my strength."

She was, in fact, confined to bed during the greater part of her life, and was subject to frequent cataleptic trances. At one time, being visited by the Abbot of Burgen while she was affected by one of these seizures, he endeavoured to move her head, but found all his exertions vain, whereupon he pronounced her to be a divine prophetess. When, however, he commanded her to arise "in the name of God," she at once left her bed as if nothing had ever ailed her. She had reached maturity before the divine character of her visions was clearly manifested. "When I was twenty-four years and seven months of age, a fiery light coming from heaven filled my brain and influenced my heart—like a fire which burns not, but warms like the sun—and suddenly I had the power of expounding the Scriptures."

She thus describes, in the letter to Wibertus, and in continuation of the paragraph which we have already quoted from it, the character of the seizures to which she was subjected:—

"During one of these states of prostration, I asked my attendant if she saw anything besides the things of this world; she replied that she did not. Then a great fear seized upon me, and I dared not open my heart to any one; but during conversation I often spoke of future events; and when the visions were strong upon me, I said things which were unintelligible to those around me. When the strength of

* Quoted by Ennemoser, *History of Magic.* Bohn's Ed., vol. i. p. 93.

the vision was somewhat abated, I changed colour and began to weep, more like a child than a person of my age; and I should often have preferred to be silent had it been possible. Fear of ridicule, however, prevented my saying anything; but a noble lady with whom I was placed noticed this, and told a nun who was her friend. After the death of this lady I had visions till my fortieth year, when I was impelled, in a vision, to make known that which I saw. I communicated this to my confessor—an excellent man. He listened willingly to these strange visions, and advised me to write them down and keep them secret, till I should see what they were, and whence they came. After he perceived that they came from God, he communicated them to his abbot, and gave me his aid in these things. In the visions I understood the writings of the prophets, the evangelists, and some holy philosophers, without human assistance. I explained much in these books, although I was scarcely able to distinguish the letters; I also sang verses to the honour of God without having had any instruction in singing—having never ever learned a song. When these things became known to the church at Mayence, they declared that these visions came from God, and by the gift of prophecy. Upon this my writings were placed before the Pope Eugene, when he was at Trier, who had them read aloud before many, and then sent me a letter begging me to commit my visions to writing."*

Now there can be little question that the abnormal mental phenomena which characterized the lives of Saints Suso, Catherine of Siena, and Hildegarde, as well as the instances stated by Bede, were of a kindred nature with those which formed the substratum of Plato's opinions upon the kinship of madness and genius. There can be little question, also, from the recital we have just given, that the twofold paradox of the ancient Greek philosopher—to wit, the superiority and great good of madness as compared with sanity—flourished vigorously under another phraseology in the Middle Ages.

If we search in our own time for indications of this paradox, we need not look far. We may instance Swedenborg as an illustration of the religious phasis of the paradox. Jung-Stilling is inclined to believe that the "capability of experiencing the arrangements which are made in the world of spirits, and executed in the visible world," may be promoted by drinking ardent spirits. He tells us also that "those who possess this capability are generally simple people;" and he continues—"It again follows from hence, that a developed faculty of presentiment is by no means a quality which belongs solely to devout and pious people, or that it should be regarded as a divine gift; I take it, on the contrary, for a disease of the soul, which we ought rather to heal than promote. He that has a natural disposition for it, and then fixes his imagination

* Ennemoser, *Op. cit.*, vol. i. p. 96.

long and intensely, and therefore *magically*, upon a certain object, may at length be able, with respect to this object, to foresee things which have reference to it. Gravediggers, nurses, and such as are employed to undress and shroud the dead, watchmen, and the like, are accustomed to be continually reflecting on objects which stand in connexion with death and interment; what wonder, therefore, if their faculty of presentiment at length develop itself on these subjects;" and then he adds the remark already quoted on ardent spirits.*

Jung-Stilling's belief respecting spirituous inspiration is perfectly consistent with the teachings of Scandinavian mythology, in which mead or beer rightly stands metonymically for poetic genius. From the *Prose Edda* we learn that the dwarfs Fjalar and Galar prepared mead or beer by mixing the blood of the universal genius Kvasir with honey, and that the liquor so prepared was of such surpassing excellence that whosoever drank of it acquired the gift of song. This divine beverage was the source of all poetic genius, and it is easy to conceive how in the first place the effects of spirituous drinks gave rise to the myth, and in the second place the myth gave rise to the conception of the inspired character of tipsiness.

Mrs. Crowe considers it "worthy of observation that idiots often possess some gleams of the faculty of second sight or presentiment," and stumbling over a subjective phenomenon of vision, she is glad to receive a helping hand from the paradox which concerns us.

"All somnambules of the highest order," she writes—"and when I make use of this expression, I repeat that I do not allude to the subjects of mesmeric experiments, but to those extraordinary cases of disease, the particulars of which have been recorded by various continental physicians of eminence—all persons in that condition describe themselves as hearing and seeing, not by the ordinary organs, but by some means the idea of which they cannot convey further than that they are pervaded by light; and that this is not the *ordinary* physical light is evident, inasmuch as they generally see best in the dark—a remarkable instance of which I myself witnessed. I never had the slightest idea of this internal light till, in the way of experiment, I inhaled the sulphhuric ether; but I am now well able to conceive it; for, after first feeling an agreeable warmth pervading my limbs, my next sensation was to find myself—I cannot say in this heavenly light, for the light was in *me*—I was pervaded by it; it was not perceived by my eyes, which were closed, but perceived internally, I cannot tell how. Of what nature this heavenly light was—I cannot forbear calling it *heavenly*, for it was like nothing on earth—I know not, &c."†

* *Theory of Pneumatology.* Translated by Samuel Jackson. Lond., 1834, p. 197.
† *The Night Side of Nature.* Ed. 1853, pp. 362 and 470.

Again, Ennemoser,* with a woful waste of learning, seeks to prove the frequent development of prophetic power in many bodily affections, and particularly in cataleptic and ecstatic states and certain inflammatory diseases of the brain. He quotes with approval a case "related by Hunaud (*Dissert. sur les Vapeurs*) of a cataleptic girl who predicted future events, as, for instance—'I see poor Maria, who takes so much trouble about her pigs; she may do what she likes, but they will have to be thrown into the water.' The next day six of the pigs were driven home, and a servant fastened them up in a pen, as they were to be killed the next day. During the night, however, one of them went mad, having been bitten a few days before by a mad dog, and bit all the other pigs. They all had to be killed."† He also writes: "The powers of the seer are very often remarkable in insanity, and express themselves in direct or allegorical language. Claus, the fool, at Weimar, suddenly entered the privy council and exclaimed, 'There are you all, consulting about very weighty things, no doubt; but no one considers how the fire in Coburg is to be extinguished.' It was afterwards discovered that a fire had been raging at the very time in Coburg."‡

Ennemoser, also, contends zealously for the supersensual character of visions, and whether they be brought about by bodily disorder, by magical operations, or by divine interposition, he links them all together, as well as the power of prophesying, and solves all difficulties with MAGNETISM. This is the key which unlocks all the mysteries of ancient and modern superstition, all the intricacies of magic, and explains why the ABNORMAL is of greater nobility than the NORMAL. He carefully describes, however, the differences which exist between the visions of the inspired seer and those of a lower grade, produced by human means, and he is careful to isolate the dignity of the Christ, and to reprove those who have reduced the God-man to an ingenious magnetiser. He writes:—

The visions of the magicians are, even in the highest stages of enthusiasm, merely shadowy reflections, surrounded by which, the world, with its significations and even its inner constitution, may be seen by him; but the lips are silent in the intoxication of ecstasy and the dazzling light of his *pathologic self-illumination.*§ On this account, the many phantasmagoria of truth and falsehood; the changing pictures of the imagination, and the feelings, in disordered ranks and inharmonic shapes; the wanderings and convulsions of the mind and body. Their visions are not aways to be relied upon, neither are they always understood. In the prophets, visions are the reflection and illumination of a divine gentle radiance on the mirror of their pure

* *History of Magic, passim.*

§ The italics are ours.

soul, which retains its whole individuality, and never forgets its perfect dependence and connexion with God and the outer world. The contents of these visions are the common circumstances of life—religious as well as civil; the words are teachings of truth, given clearly and intelligibly to all men and ages. The prophet neither seeks nor finds happiness in the state of ecstasy, but in his divine vocation to spread the word of God; not in an exclusive contempt, but in the instructing and active working among his brethren.* . . . . If we know Christ as the evangelists and apostles represent him, if we pay attention to the events before and after the advent of Christ, we shall not find it difficult to gain proper views upon the worth and intention of magnetism on the one side, and of the being and dignity of Christ as a divine manifestation and as a miracle in nature on the other."†

Thus, then, we find that Plato's twofold paradox exists among us in all its entirety, and a little reflection will show that it has a lusty life. For recent generations have simply cast aside the form and not the substance of the paradox as it existed in the "antique time." The supposititious entities of animal magnetism and odylism have been substituted for a divine afflatus; and a pseudo-philosophical has taken the place of a superstitious terminology, and that is all. The paradox remains, is dandled in the arms and hugged to the souls of many.

There is this difference, however, between the ancient and the modern supporters of the paradox: the former represented the highest philosophy of their period, the latter the most eccentric reasoning of ours.

At this point, six months ago, we might have left the question, but now the paradox has entered upon another phase of its existence. It claims to be admitted again within the legitimate bounds of science, and M. Moreau (de Tours) comes forward as its godfather. In a recently published work‡ he advances the following argument:—

"The mental qualities which cause a man to distinguish himself above other men, by the originality of his thoughts and conceptions, by the eccentricity, or the energy of his affective faculties, or by the transcendence of his intellectual faculties, have their source in the same organic conditions as the different moral disorders, of which *insanity* and *idiocy* are the most complete expression."

Let it be conceded that the explanations which have been offered at various times concerning the inequalities of intellectual capacity which exist among men have been insufficient. Neither education, considered in its widest sense, nor the configuration of the

* *Op. cit.* Vol. i. p. 90. † *Ib.* p. 336.

‡ *La Psychologie Morbide dans ses Rapports avec la Philosophie de l'Histoire ou de l'Influence de Névropathies sur le Dynamisme Intellectuel.* Par le Docteur J. Moreau (de Tours), Médecin de l'Hospice de Bicêtre. Paris, 1859, pp. 576.

cranium, nor the developmeut of the brain, nor the number, direction, or extent of its convolutions can satisfactorily solve the problem. Dr. Moreau seeks its solution in the diseased organism.

"The organization, under the influence of causes which we shall study in due time, does not pass hastily, and as it were with a leap, from the normal to the abnormal state, from the state of health to that of sickness. It begins by undergoing intimate and profound changes, which are as the first vibrations impressed by the morbific causes. These causes, in nearly every case (in heritage, for example), have acted from the first formation of the human being, since their apparent effects are manifested at a subsequent date.

"In pathology, this state of the organism is called predisposition. It is this very state that we consider as the origin, the primordial and generative fact of the phenomena of ideogeny (des phénomènes d'ideogenie) which are the object of our studies; a fact half physiological, half pathological, of which *insanity* and *idiocy*, when it refers to the nervous system in general, and the brain in particular, express the highest degree of development."—(p. 29.)

Now, according to Dr. Moreau, "every affection of the nervous system is identical as to essential character with the cerebral disorders of which the words *insanity* and *idiocy* sum up the innumerable symptomatological varieties.—"(p. 570.)

All affections of the nervous system are, indeed, linked together. They have the same predisposing causes, the same hereditary antecedents, and they arise from the same pathological source. That source is the morbid predisposition already referred to, which Dr. Moreau regards as a species of nervous orgasm or erethism from which at any moment may be educed, by the action of an occasional cause, the phenomena of insanity, idiocy, or any other of the neuroses, according as the orgasm was realized in one or other portion of, or generally diffused throughout the nervous system.

It may be objected, however, that—

"In form and sensible character, idiocy and insanity differ so profoundly the one from the other, that it is difficult to believe that these two maladies can have the same origin, and depend upon the same causes. On the one side, there is excess of vitality, exaggeration, perturbation of the intellectual and motor powers; on the other, decrease, sometimes almost complete annihilation of these powers, and of this vitality. In what manner, then, can effects so different arise from one and the same source?

"The difficulty is only apparent. Variety of effects does not imply difference in the nature of the cause; it depends upon this that the cause exercises its action at different epochs of the physical and moral development of the human being modified by it. Before birth, upon the fœtus, the pathogenic cause arrests more or less completely the evolution of those faculties, the whole of which constitute what has been termed the life of relation. We can conceive, then, that its influence diminishes in proportion as it is exercised at an epoch more remote from birth, and that its effects approximate more to insanity properly so called.

"Thus then, in the presence of the facts that hereditary transmission reveals to us, concerning the truly prodigious quantity of nervous states of every kind that are observed among the ancestors of idiots and imbeciles, as well as in the lineage of lunatics and epileptics, it is impossible, in spite of difference in symptomatological characters, not to admit that idiots and imbeciles, lunatics and epileptics, are born and developed under the same influences, as effects of one cause, as branches of one and the same trunk.—(p. 54.)

In the next place, Dr. Moreau endeavours to show that the scrofulous and rachitic constitutions dominate in the majority of idiots, and that they are linked at many points to other neuroses. He also holds that these constitutions combined with the nervous acquire an importance even superior to that of insanity, since from their transmission by heritage arises that vast mass of imbeciles, half-witted, animalized individuals, who are governed almost solely by their passions, and who form the substratum of the criminal and vicious classes.

Further, he insists upon the consanguinity of scrofula and rachitis, and concludes that—

"Individuals in whom the scrofulous and rachitic diatheses exist, whether from hereditary predisposition alone, or from the confirmed disease, are, both in a physical and moral point of view, in conditions of organization and vitality analogous, if not identical, with those of idiots and imbeciles. . . . *Lunatics* and *idiots*, the *scrofulous* and *rachitic*, in virtue of their common origin and of certain physical and moral characters, ought to be considered as children of one and the same family—divers branches of one and the same trunk."—(p. 99.)

The influence exercised upon the intellectual operations by those pathological states of which *idiocy* and *insanity* are the phenomenal expression, and to which in the preceding generalization *scrofula* and rachitis have been joined, and the laws in virtue of which this influence is exercised, are next considered by Dr. Moreau. We need only quote here, however, an opinion of that gentleman concerning the mental phenomena, an application of which is sought in this part of his treatise. He writes:—

"In all circumstances these phenomena emanate from the same focus—that is to say, from the morbidly exalted *nevrosité*, as well under the dominance of the law of inneity (for example, in the case where the nervous state is met with in the individual only) as under the dominance of the law of heritage (when this state shows itself among the parents)."—(p. 104.)

This then is the pathological substratum of Dr. Moreau's thesis, and upon this foundation he enacts the propositions which more immediately interest us. "It now remains for us to inquire," he writes, "if, as I have already asserted, the neuroses—idiocy and insanity in particular—are not the true source of pre-eminence of the intellectual faculties."

In all that he has said in the preceding part of his work a primary object has been to prove that the neuroses always, and under all circumstances, are characterized by exaltation of the vital properties, or, to adopt a less vague and less hypothetical expression, by an excess of life."—(p. 383.)

This, he conceives, is evident in chronic or acute, partial or general delirium; in the early phenomena of accidental idiocy; in the mental precocity and exuberant vitality which mark the first stages of scrofula and rachitis; in the convulsive movements of every degree which are peculiar to many neuroses (epilepsy, hysteria, &c.), all these phenomena are indicative of an excess of vitality.

If it be objected that at a certain stage of the neuropathic affection the phenomena appear to be in direct opposition to this assertion, as for example, in dementia and in stupor, in which conditions a distinct enfeeblement of the vital properties is observed, it is to be remarked that "these phenomena are even the surest indication of the excess of vitality which had existed at the commencement of the malady, an excess which had ended by breaking the wheels of the machine, as an exaggerated tension forcibly breaks a spring."—(p. 383.)

Again, perversion of a faculty or function is not to be confounded with feebleness, or disturbance with debility. The movements of the soul may lack co-ordination, and yet there may be no enfeeblement of the vital principle.

"It results from this that the neuropathic state imports necessarily into the organism a new element of life, gives an unaccustomed impulse to the play of the organs or organic media specially charged with nervous manifestations, whence hyper-activity of soul, when the intellectual apparatus is most particularly affected; hyper-activity of movement when the muscular—a hyper-activity which if it become exaggerated above what comports with the laws of the economy, degenerates into insanity in the first case, and convulsions in the second."—(p. 384.)

These things being premised, we are in a position, Dr. Moreau thinks, to comprehend that no contradiction of terms is involved in the affirmation that a disturbed state of the intellectual faculties can become, by hereditary transmission, the source of a mental condition essentially opposed—that delirium and genius have, indeed, common roots. Recall, for a moment, the psychical and physical characters of mental alienation, and of all nervous disorders; "the functional hyper-activity which necessarily flows from these affections, and of which delirium, exaltation, and incoherence of ideas, versatility and violence of sentiment, are the exterior reflexion; and it will be comprehended that this assimilation (in reference to their origin and physiological substratum) of insanity and of the most sublime qualities of the intelligence, is perfectly legitimate, more than legitimate, necessary."—(p. 384.)

We shall now be prepared to understand the genesis of genius according to Dr. Moreau. By the term hereditary predisposition, is implied an organic state which contains potentially the malady of which it is too often the sad precursor. And this idea, Dr. Moreau insists, includes implicitly another, to wit—"that of hyper-excitation, of an increase of vitality in the system of organs charged with nervous manifestations."

"This hyper-excitation constitutes in our eyes, and for pathologists who have studied the question, the first period of disease; from whatever source this arises, whether from deleterious agents introduced into the economy, or from deleterious principles developed spontaneously in the tissues themselves.

"Placed in these special conditions the organs act necessarily with a force that they have not in their ordinary state, as a machine of which the motive springs have received increased tension.

"Now, this functional hyper-activity, what can it be when it acts upon the organ charged with the manifestations of the thinking faculty? By what signs is it manifested exteriorly?

"Evidently by ideas more numerous, by greater rapidity of conception, by increase of activity and of spontaneity in the imagination, by greater originality in the character of the thoughts, and in the mental combinations, greater novelty and variety in the associations of ideas, more vivacity in the memory and audacity in the workings of the imagination, more mobility, and also more energy and more abandonment in the instincts, the affections, &c.

"For the rest, in producing this hyper-excitation in the nervous functions, heritage comports itself in the same manner as all the agents which modify the general innervation.

"If the excitation passes beyond certain limits; if, by the violence of its action, it dominates the *Me*, that is to say, the interior principle destined to bind together, to co-ordinate the action of the different intellectual powers, in place of heightening the qualities of the mind and communicating to them an unaccustomed brilliancy, it leads directly to madness.

"Certainly, I hasten to remark, lest my thought should be overstrained by any one, it would be a great error to seek solely in the organic conditions of which I speak for the source of genius, or simply of a certain superiority of the intellectual faculties. There rests always *a something unknown* (*quid divinum*) to disengage; else genius would be as common as it is rare, by the facility with which every one would be able to procure it by the aid of some cerebral excitants.

"But it is equally certain that these conditions favour powerfully the fulfilment of the intellectual functions.

"Two conditions, in effect, appear fundamentally necessary for the perfect play of the cerebral organism: the first, the most important without doubt, and which may be termed *the* essential condition (*condition par excellence*), comprehends certain intrinsic qualities which belong to the essence even of the organization; the second is related to a certain physiological state, which is to the accomplishment of the intellectual functions that which the stimulus produced by the oxygenation of the venous blood is to the accomplishment of the vital in general.

"This second condition is that which shows itself most plainly through the influence of hereditary transmission, and especially through the means of foreign agents, whether physical or moral. . . . . . . . . With these reservations we believe that no one can refuse to regard cerebral disorders as an hereditary condition apt to favour the development of the intellectual faculties."—(pp. 398-99.)

We need not cull from Dr. Moreau's thesis any of the examples which he thinks may be derived from the results of enthusiasm, of certain agents capable of acting upon the nervous system, of certain pathological states of the brain (simple or in febrile affections), and from the psychical manifestations at times observed in the death-agony, illustrative of the truthfulness of his propositions. The examples we have already quoted from Bede, the lives of certain saints, and other sources, will suffice, as they are of the same class as those which Dr. Moreau makes use of. He thinks that the illustrations he cites amply justify the assertion that maladies of the nervous system favour powerfully the development of the intelligence. He reminds us also that he has shown reasons for a like asseveration respecting scrofulous and rachitic affections; wherefore he concludes that in a given case the intellectual functions would be most perfectly performed when these different morbid states are found united in the same individual—

"That is to say, when the subject is of a constitution at one and the same time rachitico-scrofulous and neuropathic; in other words, when by his constitution he touches *idiocy* on the one hand, and *madness* on the other.

"All this implies necessarily another proposition—to wit, whenever the intellectual faculties are sure to be elevated above the common level, in those cases especially where they attain a degree of energy every way exceptional, we may be certain that the neuropathic state, under one or other form, will have influenced the organ of thought either idiopathically or hereditarily; that is to say, sometimes in virtue of the law of inneity, sometimes in virtue of the law of imitation. This leads again to the conclusion that exceptional men have the same conditions of origin or of temperament as the insane and idiots."—(p. 463).

We have but to add one or two of Dr. Moreau's ultimate corollaries to complete our slight history of the twofold paradox which has been our theme.

1. "*Genius*—that is to say, the highest expression, the *ne plus ultra* of intellectual activity, is a *neurosis*."

Why not? he asks. We may, he tells us, accept the definition very well, "if we do not attach to the word neurosis a signification as absolute as when it is applied to the different modalities of the nervous organs, and in making it simply the synonyme of exaltation (we do not say disorder, perturbation) of the intellectual faculties." In fact, we are to use the word not in its legitimate signification, but in one given to it for the occasion! Thus:—

"The word neurosis indicates then a particular disposition of these faculties, a disposition participating always of the physiological state, but overstepping already the limits of that state and touching the opposite one, which is so well explained by the morbid nature of its origin. . . . . . The word neurosis expresses simply a special state of the brain corresponding to that disposition of the intellectual power . . that is termed genius. In other terms, genius, like every other disposition of the intellectual dynamism, has necessarily its material *substratum;* this substratum is a semi-morbid state of the brain, a true nervous erethism, of which the source is nevertheless well known to us."—(p. 465.)

2. "The old maxim 'Mens *sana* in corpore *sano*,' is false. Precisely the reverse of this holds good.

—"In truth, if the normal state of the organism accord generally with the normal action of the thinking faculty, never, in any case, or only exceptionally, is the intelligence seen to elevate itself above the common level of that which is called mediocrity, as much in an affective as in an intellectual point of view, properly so called.

"In these conditions, man might be endowed with a right sense, a judgment more or less severe, a certain degree of imagination, his passions would be moderate; always master of himself, he would unquestionably practise better than any one the doctrine of interest. He would never be a great criminal, neither would he ever be a great man of probity, nor even be attacked with that *mental malady* that is called *genius;** in short, under any circumstances, he would never be noted among privileged beings."—(p. 468.)

3. "Madness and genius are congeners, *in radice conveniunt.*"—(p. 493.)

Here, then, we have returned to the very point from which we

* Lamartine.

at first started—the twofold paradox enunciated by Plato, that madness is of greater nobility than sanity; and that a distempered mind, so far from being an unmitigated evil, is, in fact, a notable blessing.

Nay, even the very language in which Dr. Moreau expresses the essential character of the pathological notions upon which his version of the paradox is founded, may be paralleled among the ancient philosophers. Thus Dr. Moreau writes:—

"All intelligence may be classed successfully, and in an uninterrupted manner, upon the different degrees of a scale of which the inferior extremity is occupied by the *idiot*, by imperfect human beings, reduced, in their moral existence, to incomplete sensations or perceptions; and the summit by the maniac, a prey to the most violent exaltation. I distinguish indistinctly the place which that which is called reason occupies between these two extremes; if I mount a degree higher, I find a mental condition, a peculiar disposition of spirit which is certainly already something more than reason, but which is still not mania, it is *excitation*."

And so Cicero writes in his treatise on *Divination:*

"As men's minds were often seen to be *excited* in two manners, without any rules of reason or science, by their own uncontrollable and free notion, being sometimes under the influence of frenzy, and at others under that of dreams."—(§. 2.)

Are we then to admit that the relations which exist between genius and insanity are so inextricable that from whatever point of view we observe them, however thoroughly they may be analysed, we are compelled in expressing them to have recourse to a paradox? Is it true that the paradox of which we have sketched the history has a legitimate claim to be admitted within the boundaries of psychological science? We believe not.

It is obvious that if we use expressions which tend to confound together two different classes of phenomena, nothing but confusion can result. Gradually and insensibly, as morbid may shade off into healthy states, or healthy into morbid; nevertheless, the two states exist. The limitation of our present information respecting their points of departure the one from the other, affords no justification for the adoption of any hypotheses which confound the one state with the other at the root. Speculation of this kind in place of aiding, impedes research, by substituting foregone and hypothetical conclusions for suggestive observation.

It is not a novel thing to use pathological phenomena to aid in the elucidation of physiological; but it is in some sort new to use, as M. Moreau does, pathological states as normal standards of comparison. Morbid conditions of the body form most valuable and even necessary aids to the physiologist in his attempts to unravel the mysteries of the animal economy, but only in so far as they can be referred to a given standard. The terms normal and abnormal as applied to certain collective phenomena are tolerably well understood, and the phenomena to which they apply are not difficult to be apprehended so long as the words are made use of simply as concrete terms. It is well also to remember that these terms are relative as well in their signification as their application. The normal condition of one man is not that of another, but a *mean* notion of normal action is obtainable, and is always made use of, expressed or implied, and has the same relation to questions of health and disease as the *mean* in every question of physical science. This mean notion must of necessity be our standard of judgment as to the normal state of the body or any of its functions. It must be our point of departure in reasoning upon health and disease. The very term abnormal mean would be a contradiction.

Again, this *mean* spoken of is not an abstract, but a concrete idea. It is derived from an experiential judgment of the most perfect modes and conditions of action of the whole or any one of the functions of the body. The moment we regard normal and abnormal phenomena from an abstract point of view, that moment we plunge into a maze of inutile speculation, and ingenuity usurps the place of observation. Normal and abnormal are words clear, distinct, and comprehensible as concrete terms; vague, inexplicable, unmeaning as abstract.

Now it has been necessary to premise the signification we attach to two technical words in common use, because we believe that it has been in no small degree from laxity, or vagueness, or peculiarity in the use of these and other terms that Dr. Moreau has stumbled into paradoxes.

Thus, he conceives that the very essence of the organic condition transmitted by heritage, that is hereditary predisposition (the latter word being used in its pathological sense), is hyper-excitation. This state he describes "as an increase of vitality in the system of organs charged with nervous manifestations."—(p. 397.) Elsewhere he also uses the term as synonymous with "increase of life." Note carefully the phraseology of the definition, and the term defined. Excitation and excitement are common phrases applied to certain well-known categories of phenomena. Dr. Moreau uses the term to express a pathological state and a theory of morbid generation; yet he gives as an equivalent term an increase of vitality simply. He speaks of *degree* of action, but he implies changed *quality* of action. He tells us that "predisposition" is a pathological state (p. 30); he uses the term hyper-excitation in a pathological sense; in both instances modification of the *quality* of action is conveyed, and yet he gives as a synonymous expression increase of vitality—a change of *degree* only. *Excitation* with Dr.

Moreau is the name of a theory, and not the expression of a fact. Throughout the whole of his argument a theoretical and abstract idea is to be attached to the word excitation; but he nevertheless makes use of it in the common fashion, and as if the ordinary signification belonged to it, hence a never-ending source of confusion. Indeed, the proposition that hyper-excitation, a pathological state, is simply an increase of vitality, arises from the same laxity of phraseology and expression which has led to the conclusion that genius and insanity are congenerous.

To adopt any word in common use to signify certain well-defined phenomena, as the exponent of a particular theory, cannot be too strongly condemned. Vagueness and confusion must inevitably result from acting thus; and this is not the first time that the importation of the word excitation, or one of its congeners, into science as the representative of a theory has done mischief.

We have already seen in what manner Dr. Moreau, under the stress of his theory, has had to deal with the word neurosis. In his notions respecting the manifestations of the normal action of the mind, it has been necessary also for the integrity of his belief, that he should attach to them the idea of mediocrity, forgetting the fact that the notions from which he starts are *average* ones, and not *absolute*, and consequently that the idea of mediocrity could only have an average application. Hence the very foundation of his denial of the axiom, "Mens *sana* in corpore *sano*," is an assumption, necessary for his theory, not for the facts.

Dr. Moreau admits that morbid action will have no effect in eliminating genius, unless there be the prior capacity—the prior *something* from which genius springs. Indeed, the morbid action is simply a cause favouring the development of genius; but, according to him, so important a cause that without it the intellect would never reach its highest development. It perfects the mental soil and determines the choicest intellectual bloom, or, curious antithesis, the uttermost moral perversion. Is physiology so meagre in its information on this subject that even for a moment it is necessary we should fall into this wretched and humiliating paradox? Surely not.

We are taught, and to us seemingly on incontrovertible grounds, that the substratum of all mental action is automatic. The laws governing cerebral action are precisely similar to those governing the action of other nervous centres, with something besides. We can trace in the whole class of mental operations automatic action of a nature analogous to that exhibited by the brute creation, but with the addition of an intelligential volition which is peculiar to man. This intelligential volition constitutes the only criterion we possess of the normal or abnormal state of the human mind.

We are taught also that the very groundwork of our highest intellectual manifestations is instinctive. Our sense of beauty, of harmony, of truth, of right, are developed spontaneously within us; they are intuitive. Now it is in the primarily instinctive character of these intuitions, and the automatic nature, as well natural and acquired, of many mental operations, that the explanation of the phenomena which have led to the paradox reproduced by Dr. Moreau consists. The extraordinary development of one or other of the mental intuitions as exemplified in some forms of genius, and occasionally in somnambulism and dreaming, as well as the unusual muscular power, or amazing precision of its action witnessed in certain bodily affections, are familiarly spoken of as higher manifestations of the mental or motor powers. That they are indications of *morbidly* increased action may be admitted, but that that action is increased in reference to the normal manifestations of the faculties named we deny. With man the standard of judgment is intelligential volition, not automatic action. Witness the automatic operation of the mind in the dreamer; of the mind and muscular system in the somnambulist; of the æsthetic gifts of certain eccentric geniuses; and what do we behold but instinctive actions of the same class as those observed in the brute, the bird or the insect, but not as in these creatures curbed and directed to a useful end by a Higher Will, but astray, erratic, anomalous from the lack of the deputed will. We witness the possible capabilities, the potential powers of the brain and nervous system in these cases, but the culmination of mental action, the intelligent directive power, is wanting. Compare the visions of the ecstatic with the lucubrations of a Butler; the automatic movements of a somnambulist with the trained action of the prestidigitator, the acrobat, the fingers of the musician, and of many a craftsman; the genius of the semi-madman with his shattered volitional control. In the whole of the former states we behold a lower grade of mental action in man as man; we see automatism usurping the place of intelligential volition; *instinct* superseding *insight*.

Now we affirm that the *quasi*-high intellectual states which are observed in certain morbid conditions of the nervous system, are invariably characterized by a preponderance of the automatic over the ratiocinative actions of the brain. That there is with these states a greater or less loss of that co-ordination of the faculties which is necessary for the most perfect intellectual action. But to describe genius of this stamp as the highest manifestation of the intellect, is simply a perversion of terms. Wherever genius of any form is found associated with a morbid condition of the nervous system, there it may be predicated we shall find a more or less manifest determination from the normal action of the intellect in its entirety. In no respect is this more clearly remarked than in the preponderance of impulse over motive, which, as Coleridge remarks:—

"Though no part of genius, is too often its accompaniment. For the man of genius lives in continued hostility to prudence, or banishes it altogether, and thus deprives virtue of her guide and guardian, her prime functionary, yea, the very organ of her outward life. Hence a benevolence that squanders its shafts and still misses its aim, or resembles the charmed bullet that, levelled at the wolf, brings down the shepherd. Hence desultoriness, extremes, exhaustion—

And thereof cometh in the end despondency and madness!

Let it not be forgotten, however, that these evils are the disease of the man, while the records of biography furnish ample proof that genius, in the higher degree, acts as a preservative against them; more remarkably, and in more frequent instances, when the imagination and preconstructive power have taken a scientific or philosophic direction, as in Plato—indeed in almost all the first-rate philosophers, in Kepler, Milton, Boyle, Newton, Leibnitz, and Berkeley."*

Concede Dr. Moreau's category of theories by means of which he arrives at the conclusion that idiocy, insanity, scrofula, rachitis, the neuroses, and genius are congenerous; concede to him also that this of necessity leads to the proposition, that wherever the intellectual faculties are raised above the common level, it indicates a morbid condition of the nervous system; concede these things, and it would of necessity follow that Swift's satirical demonstration that madness is the source of all human genius and of all the institutions of the universe† becomes a profound truth. As such it is regarded by Dr. Moreau, who mentions it as an *instinctive* appreciation of the "truths" for which he combats. Need we say more?

We may have to widen our notions of the extent to which morbid action affects the mind in persons not regarded as insane, but upon a question of such great import in its social bearings, mere speculation, however ingenious, is to be reprobated, and we have a right to demand rigid observation and research. There is much in Dr. Moreau's work on the hereditary transmission of insanity, and on *quasi*-insane states of the mind, which, denuded of his peculiar theories, are of great value, and we may at another time examine these portions of his work apart. Now, however, we have simply to deal with the paradoxical argument he advances, and we much fear that notwithstanding the learning he has lavished upon it, the book in which it is contained will be distinguished mainly as one of the curiosities of psychological literature.

With Dr. Moreau's work terminates our historical sketch of a curious psychological paradox—a paradox which, in its newest form, is closely paralleled by one of Clown Touchstone's

* *The Friend*, Essay i.

† *Tale of a Tub.*

logical exercitations, when seeking to prove that Corin was damned for lack of good breeding, he never having been at court. To this conclusion Corin demurred :—

*Corin.* . . . . . You told me you salute not at the Court, but you kiss your hands ; that courtesy would be uncleanly, if courtiers were shepherds.

*Touch.* Instance, briefly ; come, instance.

*Corin.* Why, we are still handling our ewes ; and their fells, you know, are greasy.

*Touch.* Why, do not your courtiers' hands sweat ? And is not the grease of a mutton as wholesome as the sweat of a man ? Shallow, shallow ; a better instance, I say ; come.

*Corin.* Besides, our hands are hard.

*Touch.* Your lips will feel them the sooner. Shallow, again : a more sounder instance, come.

*Corin.* And they are often tarr'd over with the surgery of our sheep ; and would you have us kiss tar ? The courtiers' hands are perfumed with civet.

*Touch.* Most shallow man ! Thou worm's-meat in respect of a good piece of flesh. Indeed ! Learn of the wise, and perpend ; civet is of baser birth than tar ; the very uncleanly flux of a cat. Mend the instance, shepherd.*

Does not Dr. Moreau's hypothesis, that genius is of baser birth than mental mediocrity, belong to the same form of ratiocination as Touchstone's depreciation of the nobility of civet ?

---

### Art. II.—HYSTERIA IN CONNEXION WITH RELIGIOUS REVIVALS.

* *As You Like It*, act iii. sc. ii.

